# Person Re-Identification Using Deep Modeling of Temporally Correlated Inertial Motion Patterns

**DOI:** 10.3390/s20030949

**Published:** 2020-02-10

**Authors:** Imad Gohar, Qaiser Riaz, Muhammad Shahzad, Muhammad Zeeshan Ul Hasnain Hashmi, Hasan Tahir, Muhammad Ehsan Ul Haq

**Affiliations:** 1 Department of Computing, School of Electrical Engineering and Computer Science, National University of Sciences and Technology (NUST), Islamabad 44000, Pakistan; igohar.mscs16seecs@seecs.edu.pk (I.G.); muhammad.shehzad@seecs.edu.pk (M.S.); mhashmi.mscs16seecs@seecs.edu.pk (M.Z.U.H.H.); hasan.tahir@seecs.edu.pk (H.T.); mhaq.mscs19seecs@seecs.edu.pk (M.E.U.H.); 2 The College of Optoelectronic Engineering, Shenzhen University, Shenzhen 518060, China

**Keywords:** deep learning, human re-identification, human-gait analysis, inertial sensors, inertial-based person re-identification, gait-based person re-ID

## Abstract

Person re-identification (re-ID) is among the essential components that play an integral role in constituting an automated surveillance environment. Majorly, the problem is tackled using data acquired from vision sensors using appearance-based features, which are strongly dependent on visual cues such as color, texture, etc., consequently limiting the precise re-identification of an individual. To overcome such strong dependence on visual features, many researchers have tackled the re-identification problem using human gait, which is believed to be unique and provide a distinctive biometric signature that is particularly suitable for re-ID in uncontrolled environments. However, image-based gait analysis often fails to extract quality measurements of an individual’s motion patterns owing to problems related to variations in viewpoint, illumination (daylight), clothing, worn accessories, etc. To this end, in contrast to relying on image-based motion measurement, this paper demonstrates the potential to re-identify an individual using inertial measurements units (IMU) based on two common sensors, namely gyroscope and accelerometer. The experiment was carried out over data acquired using smartphones and wearable IMUs from a total of 86 randomly selected individuals including 49 males and 37 females between the ages of 17 and 72 years. The data signals were first segmented into single steps and strides, which were separately fed to train a sequential deep recurrent neural network to capture implicit arbitrary long-term temporal dependencies. The experimental setup was devised in a fashion to train the network on all the subjects using data related to half of the step and stride sequences only while the inference was performed on the remaining half for the purpose of re-identification. The obtained experimental results demonstrate the potential to reliably and accurately re-identify an individual based on one’s inertial sensor data.

## 1. Introduction

**Figure 1:**
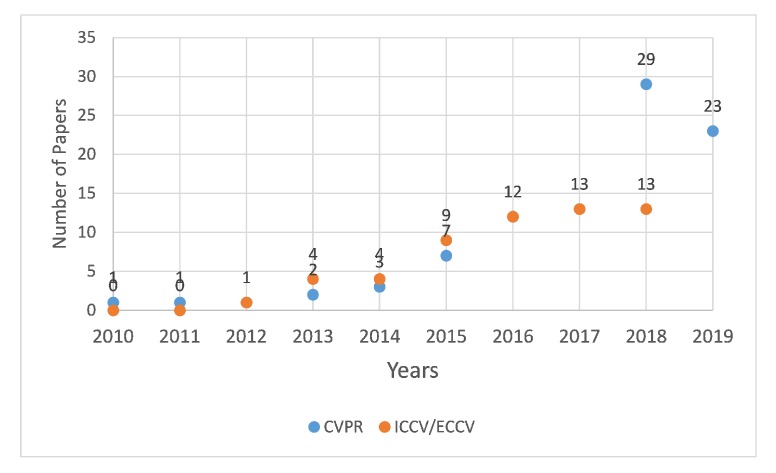
Plot depicting the increasing trend of re-ID publications in top computer vision conferences including CVPR, ICCV and ECCV.

Person re-identification (re-ID) is an important and active research area with potential applications particularly in the field of automated surveillance/monitoring, robotics, human–computer interaction, and digital forensics. The research aims to re-identify an individual by establishing correlation between features of the same person captured at different positions and time instances [1]. This is done by assigning a unique identification to individually detected persons the first time, followed by keeping track of them if identified at another location at a different time. A vast majority of the literature has addressed the person re-ID problem using vision sensors [2,3,4,5]. This is evident by the growing number of publications related to object re-ID appearing in top venues over recent years (see Figure 1).

Approaches making use of vision sensors primarily rely on appearance-based features to re-identify the same person in a non-overlapping multi-camera network. A drawback of appearance-based methods is that they make re-ID systems strongly dependent on visual cues such as color, texture, etc., consequently limiting the potential to short-term re-ID only. Similarly, in the context of practicality, these image- or features-based re-ID approaches are only suitable to work in controlled scenarios where the change in appearance (e.g., actual change in clothing, etc.) have significant impact and adversely affect their performance. Furthermore, the issues pertaining to image blurring, optical motion, effects of gender, varying illumination, etc. pose additional challenges in extracting vital features for distinctive representation resulting in the degraded re-ID accuracy.

To cope with problems presented by appearance-based solutions, several researchers have opted to incorporate other distinguishing attributes for re-identification representation. Among them, one of the most promising unique traits is the human gait. The gait of an individual is believed to be unique [6] and provides a distinctive biometric signature that is suitable for re-ID, especially in an uncontrolled environment and for smart surveillance. Research has shown the possibility of recognizing individuals solely on gait analysis (provided that the measurements of the motion patterns are unambiguously observed) [1]. However, in image-based gait analysis, it is often hard to extract quality measurements of an individual’s motion patterns in unconstrained scenarios (such as person re-ID) mainly due to problems related to scale, 3D to 2D projectile geometry, occlusions, etc. These issues are further amplified when considering conventional image related problems, which include variations in viewpoint, illumination (daylight), clothing, worn accessories, etc. These factors as a consequent limit the accurate representation of the motion patterns and therefore lead to imprecise estimation of the respective gait patterns.

With recent advancements in hardware, the use of wearable devices in estimating soft biometrics (particularly gait for behavioral analysis) has been consistently growing. Gait analysis by estimating motion patterns through wearable sensors has shown profound potential in various applications such as age estimation [7,8], gender classification [9,10], person identification [11,12], medical (diagnosis/rehabilitation) [13,14], sport activities [15], terrain classification [16], and many others. The triaxial accelerometer provides the ability to efficiently capture human motion data for gait analysis, albeit with slightly less accuracy when compared to modern motion capture devices [17,18]. The reason for this stems from the fact that these devices have the ability to capture and reconstruct human motion [19] in an inexpensive and convenient manner even with limited inertial observations [20,21]. Motion capture using inertial MEMS sensors is prone to error due to the inherent bias and the impact of external environmental noise. Despite this, an advantage of using triaxial accelerometer MEMS sensor is that it is now a standard integrated component in all modern digital devices and has a credible accuracy. This being a low-cost solution also takes away the need to mount an external sensor/device to capture motion related data. Use of inertial data obtained using smartphones has shown reasonable estimate of person’s age [8], identity [22], gait [23,24], step count [25], stride length [26], walk distance [27], etc.

This paper demonstrates the potential of re-identification of an individual by using inertial measurements units (IMU) based on two common sensors, namely gyroscope and accelerometer. Specifically, the six-axis (gyro + accelerometer) motion tracking device MPU-6500 embedded on a smartphone and a wearable inertial sensor (APDM Opal IMU) were used to capture and record the motion data of an individual. The inertial gait data, which were used in this study, were captured in one of our previous studies [8] under three different experiment setups. In total, 86 randomly selected individuals were engaged in the data acquisition process with 49 males and 37 females between the ages 17 and 72 years. The sensors (smartphones and wearable IMUs) were tightly attached on the chest of the participant with elastic belts to capture gait data. During the first step, the data signals were segmented into single strides and steps, which were individually fed to a deep neural network (DNN) architecture to learn features for re-identification. Since the data are temporally correlated, the application of Gated Recurrent Unit (GRU) [28] was appropriate instead of the conventional feed forward DNN. The GRU is a type of recurrent neural network, having two hidden layers using softmax activation functions. This allows the capture of implicit arbitrary long-term dependencies in the single stride input sequences. The experimental setup was devised in a fashion to train the network on all the subjects using data related to half of the sequences only while the inference was performed on the remaining half for the re-ID purpose. The obtained experimental results demonstrate the potential to reliably and accurately re-identify an individual based on ones inertial sensor data.

## 2. Literature Review

Human re-identification methods can be grouped into two categories, i.e., visual feature-based [2,3,29] and non-visual approaches [30,31]. Visual feature-based approaches rely on learning of features related to appearance and texture. A query-adaptive system is developed to know the importance of good and bad features for image search and person re-identification in [32]. Person Re-id is a special case of image searching where learning of similarity is done with different types of distance functions including Mahalanobis distance, Manhattan distance, Euclidean distance, etc. The Mahalanobis distance function has been used to solve the person variation problem by imposing spatial constraints [33]. A Re-id network uses a video input, extracts frames from the input video at different times and uses the extracted frames to perform Re-id. You et al. [34] extracted and combined both spacetime- and appearance-based features for solving the video-based Re-id challenges with reasonable accuracy. Ansar et al. [35] presented a two stream deep learning technique that fuses temporal and spatial information for video person re-identification removing meaningless frames via attentive pooling. The Re-id methods that are based on only appearance face the challenge of rapidly changing appearance. The problem is further compounded due to the impact of camera calibration, occlusion, illumination, deformation, and the impact of motion in scene capture.

In the context of non-visual-based approaches, the analysis of gait using inertial sensors is employed. To achieve this, one or more inertial measurement units are worn/carried to record gait patterns of the user. Accelerometers and gyroscopes play a good role in the development of motion analysis field due to their wide availability in modern devices and low-powered electromechanical sensors. They provide the potential for dynamic three-dimensional motion analysis without the constraints of a vision-based optoelectronics system [36]. Different types of inertial sensors have been experimented with in numerous studies but the reason for widespread use of accelerometer and gyroscope for human motion analysis are their small size, portability, and suitability. Optical motion capture systems for gait analysis perform well in indoor settings but fail to perform in outdoor scenarios, as the systems are sensitive to varying lighting conditions commonly witnessed in the natural environment. A suitable solution is to use inertial sensors that are low cost and equally applicable to outdoor activities as they also perform well in natural light [37].

Human identification and authentication using deep learning in an unconstrained environment with inertial sensors was previously studied by Zou et al. [38]. They recognized human gait using smartphones in an open environment. Besides this, human gait data through inertial sensors have been used to solve different problems. For instance, Qiu et al. [39] differentiated between healthy and unhealthy adults using gait data obtained through inertial sensors. An inertial motion capture system is proposed in [40] that combines visual and inertial sensors to estimate human gait with low cost and high accuracy. The proposed system solves the nonlinear optimization problem. Similarly, wearable sensors have been employed for monitoring the pathological gait disorders and diagnosis of different diseases [41]. Ahmed et al. [42] used gait samples to predict body mass index and age. A considerably alarming value of the above physiological parameters can be used to provide suitable healthcare recommendations. The increase in age variation leads to large estimation error in independent age groups. A human age estimation technique that is dependent on age-group using support vector machines and regression model was proposed by Li et al. [43] for improving the accuracy of the system. In-depth analysis of previous research has shown that Re-id is an important area that has been well explored, however, various challenges in the vision-based method limit the potential of the application. This research addressed this problem through the usage of inertial sensors for analysis of human gait and we propose human gait as a suitable candidate for person re-identification.

## 3. Methodology

This section describes the basic architecture of the proposed system for gait-based human re-identification. This includes a brief description of the gait dataset, pre-processing of the data, network architecture, and implementation details.

### 3.1. Description of the Gait Data

**Figure 2:**
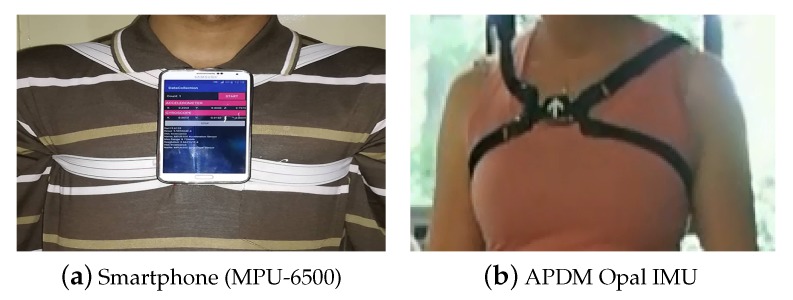
The sensors were attached tightly on the chest of the subject using elastic bands: (**a**) smartphone (MPU-6500) sensor; and (**b**) APDM Opal IMU.

**Figure 3:**
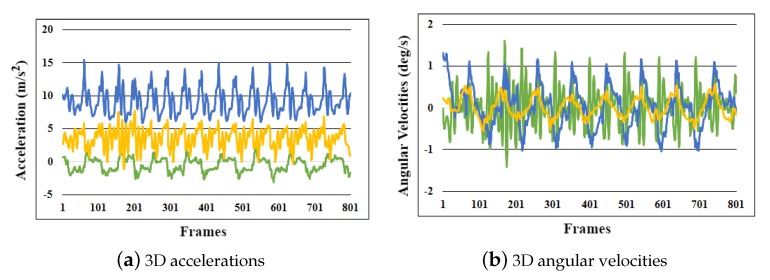
Raw low-level 6D inertial signals captured during a gait trial: (**a**) 3D accelerations; and (**b**) 3D angular velocities.

This research used the inertial gait datasets collected in one of our previous studies [8]. In total, 86 randomly selected subjects (Asians and Europeans) voluntarily participated in the data collection sessions (age (years): 33.8 ± 14.8; height (cm): 172.1 ± 8.9; male:female ratio: 49:37). The participants were instructed to walk back and forth twice in a straight line on a 10-m surface (40 m per trial). The subjects kept their shoes on in all of the trials. Three different types of experimental setups were used to capture data under varying conditions and hardware setup: Setup A (40 participants), Setup B (20 participants), and Setup C (26 participants). The subjects walked on a concrete floor (indoor) in Setups A and C, while, in Setup B, the subjects walked on six different indoor and outdoor surfaces (carpet, grass, concrete floor, soil, laminate tiles, and asphalt). With a standard sampling rate of 75 Hz, Setups A and B used a smartphone’s on-board IMU (MPU-6500) for inertial gait data collection, whereas, in Setup C, a wearable IMU (APDM Opal) was used. The sampling rate of 50–120 Hz has been extensively used in similar applications to estimate human soft biometrics [8,9,44,45,46]. Moreover, it is known that choosing a higher sampling rate increases the impact of noise and requires more processing power, which is not suitable for battery-powered/resource-constrained devices as it will result in considerably faster battery drain [47]. In the experiments, 6D accelerations and angular velocities were captured for further analysis. In all experiments, the sensors were firmly worn on the chest of with the help of a harness (Figure 2). The raw low-level 6D inertial signals recorded during a gait trial are shown in Figure 3.

It is important to note that the gait data used in this study are heterogeneous in nature which, provides several worthy benefits in human gait analysis. The subjects are demographically diverse, thus ensuring distributive justice and equipoise. Subjects intentionally modified their gait patterns (Setup A) to forge their gait; walked on a variety of surfaces, which differ in the coefficient of friction (Setup B); and used different sensor modalities (smartphones vs. wearable). These heterogeneous properties are key to correctly analyzing the correctness of the proposed person re-identification method under variations in gait for a single subject. The characteristics of the subjects who participated in experiments are shown in Table 1.

### 3.2. Signal Segmentation

Unlike many creatures in nature, human locomotion is characterized by repetitive bipedal movements of the lower extremities which form gait cycles. A gait cycle predominantly consists of a swing phase where the the foot is swung forward and a stance phase where the foot is in contact with the ground [48]. A stride is characterized as consecutive heel strikes of the same foot (swing and stance), whereas a step is described as the heel strike of one foot followed by that of the other foot (swing). During normal human walk, the stepping frequency remains between 1 and 2 Hz [49]. Since the data were recorded at a sampling rate of 75 Hz, a frequency of 100 Hz would match the normal stepping frequency of a human walk (i.e., 1–2 Hz). Similarly, a frequency of 200 Hz will match the normal stride frequency of a human walk (i.e., twice 1–2 Hz). Based on the aforementioned, the low-level inertial gait signals recorded with triaxial accelerometer and triaxial gyroscope were segmented into 100 Hz for step-based human re-identification and 200 Hz for stride-based human re-identification.

### 3.3. Proposed Architecture

This section briefly describes the proposed architecture, which is composed of gated recurrent units (GRUs)—a type of recurrent neural networks (RNNs)—to model long sequences of gait data recorded from standard human locomotion activities.

#### 3.3.1. Introduction to Gated Recurrent Unit

Sequential modeling using deep neural networks is mainly performed using RNNs. The architecture of RNNs is similar to traditional multilayer perceptrons except that they include feedback loops, which means that output of the network is fed back together with the input. This directed cycle allows RNNs to exhibit temporal behavior, meaning that they contain a kind of memory that takes into context the previous computations to determine the next ones. The RNN is inherently deep (in time) and is usually trained through a variant of the standard backpropagation algorithm, i.e., backpropagation through time [50]. However, similar to conventional deep neural network architectures, they also suffer from the gradient vanishing problem [51], especially when the input sequences are long. In such scenarios, the use of RNNs potentially poses such an issue where the back propagated gradients, which are used to update the weights of the neural network, become too small during the backpropagation process and consequently no longer contribute in learning.

**Figure 4:**
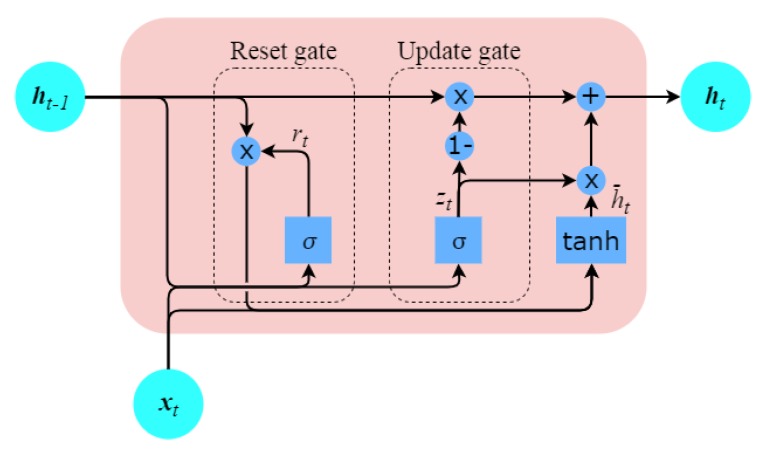
GRU with update and reset gates; the update gate decides what information to keep and what to throw away while the reset gate decides which information to keep.

To overcome the vanishing gradient problem, several sophisticated variants, e.g., the gaited recurrent units (GRUs) and long short-term memory (LSTM), are proposed. In this work, we employed GRUs to model inertial gait signals composed of essentially long 1D temporal sequences recorded while locomotion. GRUs are similar to LSTMs but have fewer tensor operations and are internally simple and a bit faster to train than LSTMs. Both LSTMs and GRUs have internal structures known as gates that regulate the flow of information. The purpose of the gates is to retain important sequences in the data while discarding others. LSTMs have three gates, namely the input, forget, and output gates, whereas the GRUs have only reset and update gates (see Figure 4). The update gate of GRUs works similarly to that of the forget gate of LSTM, where it updates the information based on its appropriateness, while the reset gate is responsible for deciding how much past information should be retained. Mathematically, these reset and update gates in GRUs can be expressed as follows [52]:

(1) $$r_{t}=sigm\left(W_{xr}x_{t}+W_{hr}h_{t-1}+b_{r}\right)$$

(2) $$z_{t}=sigm\left(W_{xz}x_{t}+W_{hz}h_{t-1}+bz\right)$$

(3) $$\tilde{h_{t}}=tanh\left(W_{xh}x_{t}+W_{hh}\left(r_{t}⊙h_{t-1}\right)+b_{h}\right)$$

(4) $$h_{t}=z_{t}⊙h_{t-1}+\left(1-z_{t}\right)⊙\tilde{h_{t}}$$

In the above equations, *r* represents the reset gate, *z* represents an updated gate, ⊙ shows element-wise multiplication, *h* is used for current, and $\bar{h}$ is used for previous activation. The activation $h_{t}$ of the GRU at time *t* is the linear interpolation between the previous activation **h_*t*−1_** and the current activation ${\bar{h}}_{t}$. The unit updates in activation or content is defined by the update gate $z_{t}$. The *sigm()* is an activation function known as sigmoid activation function, which is frequently used in GRUs to map nonlinearity. Alternate forms of this function can be created by changing $z_{t}$ and $r_{t}$.

#### 3.3.2. Model Architecture and Implementation Details

**Figure 5:**
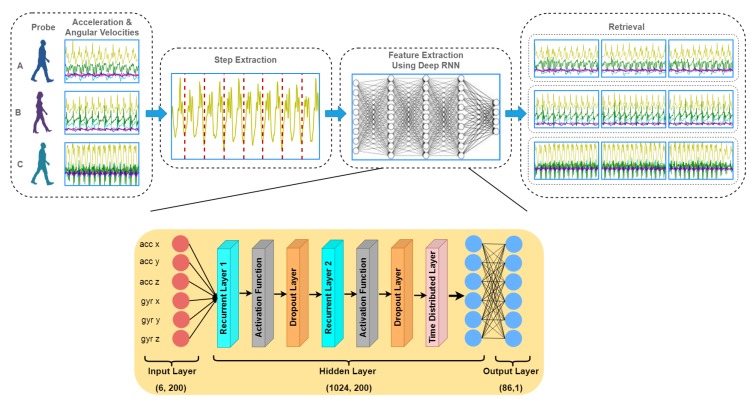
An overview of the proposed method. The gait pattern is captured using IMUs and raw signals from 6D components are used to train deep learning model.

Figure 5 illustrates the architecture details of the proposed network. The input of the model consists of raw inertial data of the accelerometer and gyroscope sensors. The stream of data is first divided into segments of 100 timesteps to extract individual steps and 200 timesteps to extract out individual strides prior to feeding them separately into the network for re-identification. The recurrent layers are bidirectional, i.e., if there are *n* number of neurons, then the output will be (n×2). In our model, we used 512 neurons per layer. Thus, the dimension of the output tensor of each recurrent layer is (1024, 100) for steps and (1024, 200) for strides, which means there are 1024 features for every timestep. Each recurrent layer is subsequently followed by an activation and a dropout layer. We employed the most commonly used activation functions in hidden layers such as *tanh* and *ReLU* (Rectified Linear Unit). Both activations provided similar results with tanh slightly outperforming ReLU. The neural networks tend to overfit, thus an effective technique to reduce the problem of overfitting is to use dropout. This simply drops a given percentage of values, so that, rather than depending too much on specific values, the model learns the generic data pattern (i.e., does a more generic prediction/inference). For the sake of data normalization, we used robust scaling instead of normal scaling (normalization). Unlike normalization, which subtracts mean and standard deviation, the robust scalar subtracts median and quartiles from the input data. The robust scalar is more robust to outliers and in the proposed algorithm it increased the model’s accuracy. We used raw signals to train the model as deep learning algorithms can extract features automatically from the input data (unlike signal smoothing/segmentation, etc. as in the case of hand crafted features approach). We employed different values for dropout and found 0.5 as the most optimal. Finally, the time distributed dense layer is added to predict one out of 86 classes for each timestep. It is essentially a fully connected layer, in which each input is connected to every neuron in the hidden layer. To take the probabilistic output, softmax activation function is used at the end. Instead of just giving one output to the whole sample with timesteps, we found that it is better to predict the output on each timestep. For this purpose, we used the time distributed layer, which enables us to obtain label for each timestep.

To train the overall network architecture, we used bidirectional *CuDNN GRU* and *tensorflow*. Categorical *cross entropy* loss function along with *adam* optimizer was used for training the network. The learning rate was set to 0.001 and batch size was kept 32 and the network was trained for 30 epochs. The value of dropout layer was set to 0.5, which was employed to prevent over-fitting. With the aforementioned configurations, the overall training took approximately 1 h on a single Tesla K80 GPU equipped desktop computer with the following details: Intel Xeon CPU model E5-2620 v4 @2.10 GHz and 16 GB RAM.

## 4. Results

In this section, the results of person re-identification computed with the step data and the stride data is presented. Three datasets were created for evaluation purposes, namely hybrid gait data (smartphone and wearable combined), smartphone gait data, and wearable IMU gait data. Furthermore, the results were computed for different sensor modalities (smartphone vs. wearable IMU), applying gender and age restrictions to evaluate the effect of gender and age on person re-identification. For performance measures, the cumulative matching characteristics curve (CMC) and mean average precision (mAP) were used. CMCs are useful to find the matching accuracy; for example, if the rank 10 accuracy of the proposed model is 90%, then it means that a match will occur somewhere in the top 10 with an accuracy of 90%. The effects of age and gender restrictions are described as classification rates with the help of bar graphs.

### 4.1. Sensor Modalities

#### 4.1.1. Hybrid Gait Data

**Figure 6:**
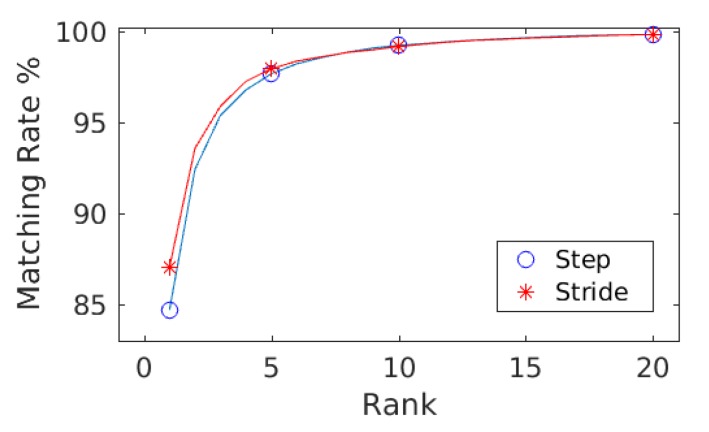
The matching rate computed against step data and stride data are shown as CMCs. Only slight variations in the matching rate are observable.

**Figure 7:**
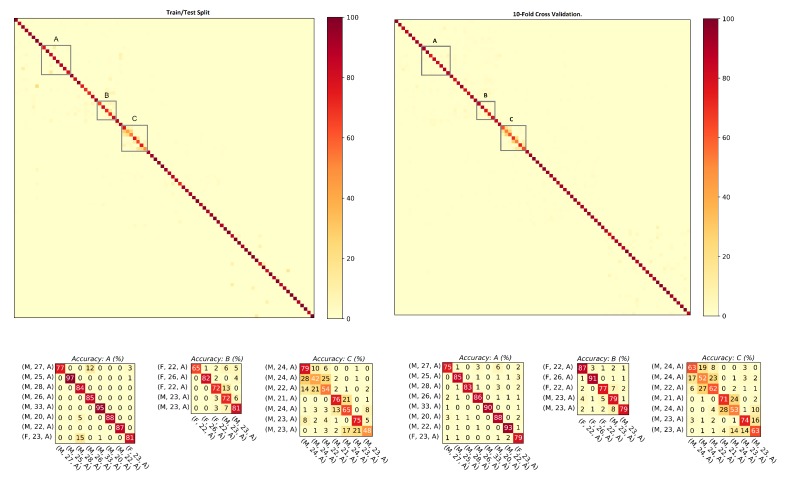
Confusion matrices computed from the steps using hybrid data: (**left**) train/test split; and (**right**) 10-fold cross validation. The convention of notations used in the axes are (gender, age, experimental setup), e.g., (F, 22, A) means that the subject is a female who is 22 years old and the data were captured under experimental Setup A. The person re-identification accuracies remained higher for most of the subjects.

**Figure 8:**
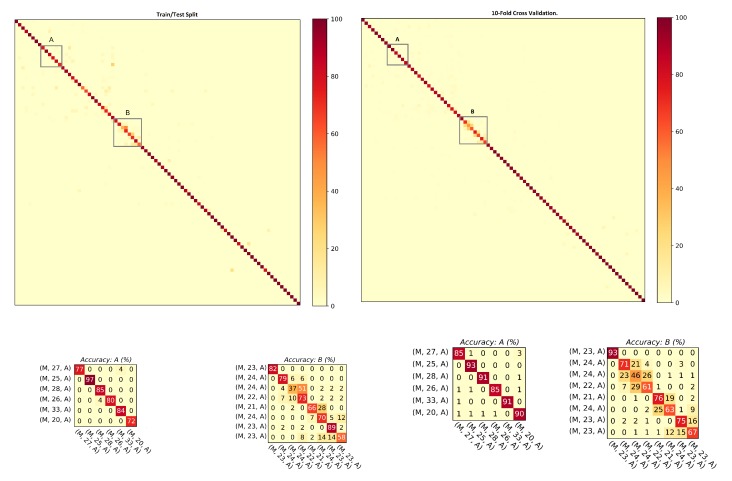
Confusion matrices computed from the strides using hybrid data: (**left**) train/test split; and (**right**) 10-fold cross validation. The convention of notations used in the axes are (gender, age, experimental setup), e.g., (M, 27, A) means that the subject is a male who is 27 years old and the data were captured under experimental Setup A. The person re-identification accuracies remained higher for most of the subjects.

The goal of evaluating results against hybrid gait data was to test the performance of the presented approach against the data collected with two different sensor modalities, i.e., smartphones’ on-board IMUs and the wearable IMUs. As explained in Section 3.1, the database is composed of 86 subjects (Asian and European) with a female to male ratio of 37:49 and 252 min of gait data recorded. This results in 1,134,000 frames, 19,614 steps, and 9807 strides of gait data. The ranking results along with mean average precision computed with the step data and the stride data are shown in Table 2. For both steps and strides, above 86% of the subjects were correctly re-identified in Rank-1, whereas above 97.50% of the subjects were correctly re-identified in Rank-5. The mean average precision remained above 90% in both cases. The comparison of the matching rate of steps and strides are presented as CMCs in Figure 6 where only slight variations in the matching rate are observable. Figure 7 shows the confusion matrices computed from the hybrid data of steps using (Figure 7, left) train/test split and (Figure 7, right) 10-fold cross validation. Similarly, Figure 8 shows the confusion matrices computed from the hybrid data of strides using (Figure 8, left) train/test split and (Figure 8, right) 10-fold cross validation. In all of the cases, the classification accuracies remained above 85%. In sum, the classification accuracies of the model trained with the stride data outperformed the model trained with step data in most cases. From the confusion matrices, it is observable that most of the misclassified subjects are 23–28 years old. The confusions are also gender dependent in most cases, where the male/female subjects are misclassified with other male/female subjects of similar ages.

#### 4.1.2. Smartphone Data

**Figure 9:**
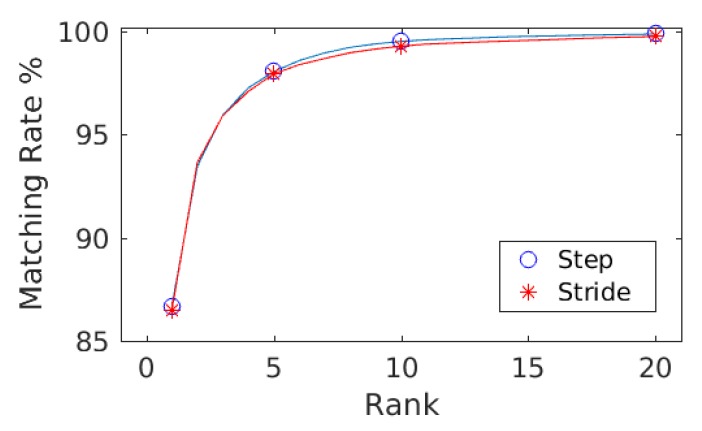
The graph shows that, for both steps and strides, above 86% of the subjects were correctly re-identified in Rank-1, whereas above 98% of the subjects were correctly re-identified in Rank-5.

**Figure 10:**
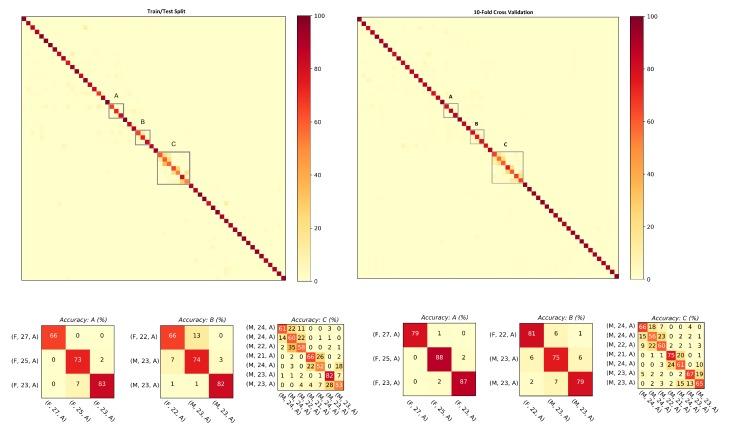
Confusion matrices computed from the steps using smartphone’s IMU data: (**left**) train/test split; and (**right**) 10-fold cross validation. The convention of notations used in the axes are (gender, age, experimental setup), e.g., (F, 22, A) means that the subject is a female who is 22 years old and the data were captured under experimental Setup A. The person re-identification accuracies remained higher for most of the subjects.

**Figure 11:**
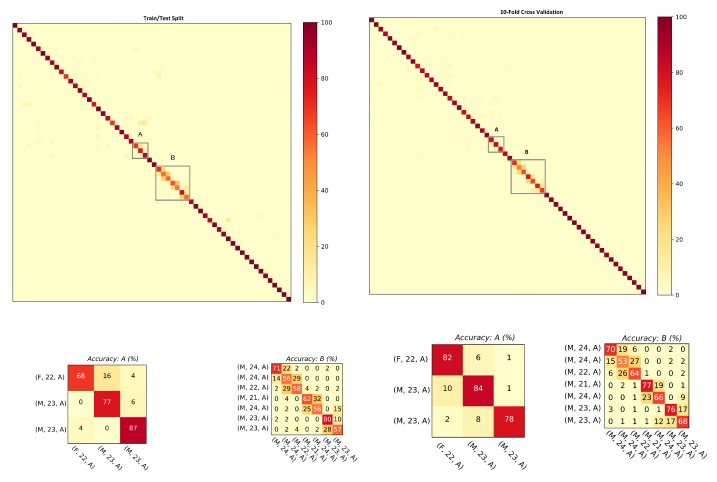
Confusion matrices computed from the strides using smartphone’s IMU data:(**left**) train/test split; and (**right**) 10-fold cross validation. The convention of notations used in the axes are (gender, age, experimental setup), e.g., (F, 22, A) means that the subject is a female who is 22 years old and the data were captured under experimental Setup A. The person re-identification accuracies remained higher for most of the subjects.

This section studies the performance of the proposed approach against the data recorded with smartphone’s on-board IMUs. The database includes 60 Asian subjects (collected under Setups A and B) with a female to male ratio of 23:37. The ranking results and mean average precision computed for the step data and the stride data are shown in Table 3. For both steps and strides, above 86% of subjects were correctly re-identified in Rank-1, whereas above 98% of subjects were correctly re-identified in Rank-5. The mean average precision remained above 91% in both cases. The comparison of the matching rate of steps and strides are presented as CMCs in Figure 9. The matching rate of steps was slightly better than that of the stride. Figure 10 shows the confusion matrices computed from the smartphone data of steps using (Figure 10, left) train/test split and (Figure 10, right) 10-fold cross validation. Similarly, Figure 11 shows the confusion matrices computed from the smartphone data of strides using (Figure 11, left) train/test split and (Figure 11, right) 10-fold cross validation. The classification rates remained above 85% in all of the cases.

As observed previously, the classification accuracies computed with the model trained on stride data predicted better than the model trained on step data. In general, the stride-based method performed better because a stride has more information (frames) than a step; however, in some rare, cases a step-based method can perform better. From the confusion matrices, it is noticeable that most of the misclassified subjects are 21–27 years old. The confusions are also gender dependent in most cases where the male/female subjects are misclassified with other male/female subjects of similar ages.

#### 4.1.3. Wearable IMU Data

**Figure 12:**
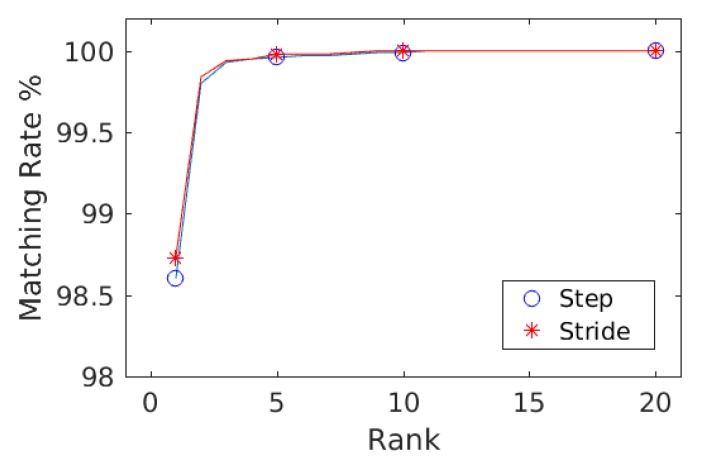
The combined curves for steps and strides is shown for comparison using wearable data.

**Figure 13:**
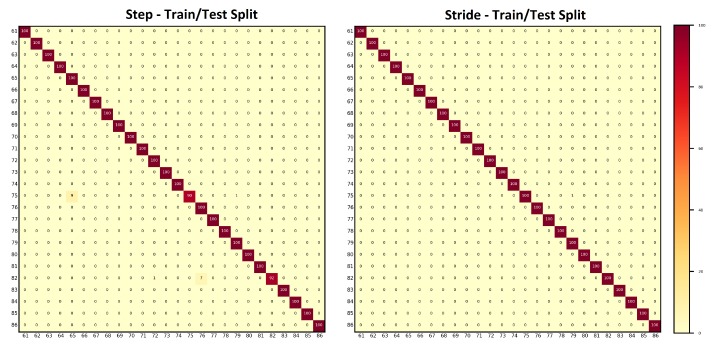
Confusion matrices computed from the wearable IMU data for: steps (**left**); and strides (**right**). The dataset consists of 26 subjects and it was collected under Setup C. The person re-identification accuracies remained higher for most of the subjects.

**Figure 14:**
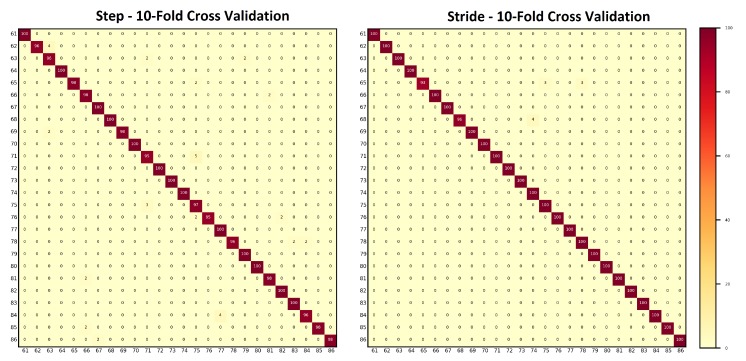
Confusion matrices computed from the wearable IMU data with 10 fold cross validation for: steps (**left**); and strides **(right**). The dataset consists of 26 subjects and it was collected under Setup C. The person re-identification accuracies remained higher for most of the subjects.

In the case of the data collected with wearable IMU, the database consists of 26 subjects (European) with a female to male ratio of 14:12. The ranking result along with mean average precision computed with the step data and the stride data are shown in Table 4 and in the form of CMCs in Figure 12. For both steps and strides, more than 98% subjects were correctly re-identified in Rank-1, whereas more than 99% subjects were correctly re-identified in Rank-5. The mean average precision remained above 99% in both cases. The comparison of matching rate of steps and strides are presented. Figure 13 shows the confusion matrices computed from the wearable data using steps (Figure 13, left) and strides (Figure 13, right) by employing train/test split validation strategy. Similarly, Figure 14 shows the confusion matrices computed from the wearable data using steps (Figure 14, left) and strides (Figure 14, right) by employing 10-fold cross validation strategy. In all cases, the person re-identification accuracies remained higher for most of the subjects.

### 4.2. Age Restrictions

#### 4.2.1. Applying Age Restrictions to Hybrid Data

**Figure 15:**
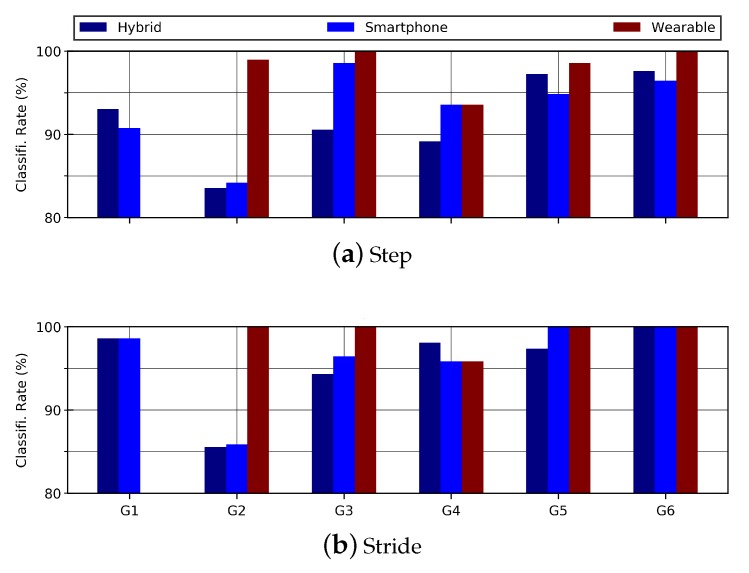
Bar graph shows the effect of age-groups on: (**a**) step data; and (**b**) stride data. In general, higher classification accuracies were achieved with the stride data.

The ages of individuals who participated in the data collection sessions were placed into six age groups: $G_{1}$: 10–19 years; $G_{2}$: 20–29 years; $G_{3}$: 30–39 years; $G_{4}$: 40–49 years; $G_{5}$: 50–59 years; and $G_{6}$: 60–79 years. The details of the number of subjects belonging to each group are shown in Table 5. The results of person re-identification on hybrid data after applying age groups restrictions are shown in Table 6 and Figure 15. For the steps, the classification accuracies remained within 83–90% for the age groups 20–29, 30–39, and 40–49. For the remaining age groups, the classification accuracies remained above 90%. In the case of strides, the classification accuracies remained at 85% and 90% for the age groups 20–29 and 30–39, respectively. For the remaining age groups, the classification accuracies remained above 90%.

#### 4.2.2. Applying Age Restrictions on Smartphone Data

The ages of subjects who participated in the data collection sessions held under experimental Setups A and B were placed into six age groups: $G_{1}$: 10–19 years; $G_{2}$: 20–29 years; $G_{3}$: 30–39 years; $G_{4}$: 40–49 years; $G_{5}$: 50–59 years; and $G_{6}$: 60–79 years. The details of the number of subjects belonging to each group are shown in Table 5. The results of person re-identification on smartphone data after applying age groups restrictions are shown in Table 7 and Figure 15. For the steps, the classification accuracies remained at 84% and 89% for the age groups 20–29 and 30–39, respectively. For the remaining age groups, the classification accuracies remained above 90%. In the case of stride data, the classification accuracy of 86% was achieved against age group 20–29. The classification accuracies for all of the remaining groups remained above 90%.

#### 4.2.3. Applying Age Restrictions on Wearable Data

The ages of subjects participated in the data collection sessions held under experimental Setup C were grouped into five age groups: $G_{1}$: 20–29 years; $G_{2}$: 30–39 years; $G_{3}$: 40–49 years; $G_{4}$: 50–59 years; and $G_{5}$: 60–79 years. The details of the number of subjects belonging to each group are shown in Table 5. The results of person re-identification on wearable data after applying age groups restrictions are shown in Table 8 and Figure 15. The classification accuracies remained above 90% for both models trained with the step data and the stride data. In general, the model trained with the stride data produced better predictions.

### 4.3. Gender Restrictions

#### 4.3.1. Applying Gender Restrictions on Hybrid Data

**Figure 16:**
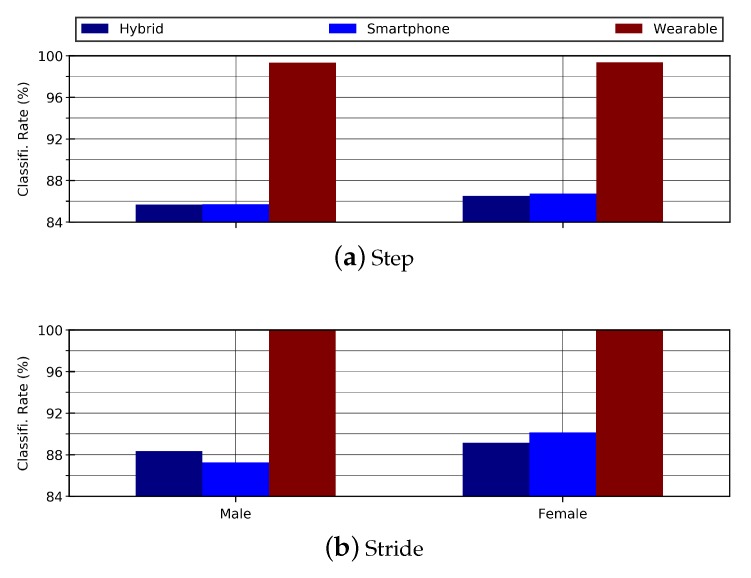
This bar graph shows the effect of gender on steps and strides for hybrid data.

The results ertr evaluated to analyze the effect of gender on person re-identification. The hybrid data include a male to female ratio of 48:38. To analyze the effect of gender, the models were trained and tested for each gender separately. On the step data, the classification accuracies of female and male subjects remained at 86.50% and 85.67%, respectively. On the stride data, the classification accuracies were slightly better than those of the step data. Classification accuracies of 89.12% for female group and 88.34% for male group were observed, as shown in Table 9 and Figure 16.

#### 4.3.2. Applying Gender Restrictions on Smartphone Data

The results of gender restrictions on hybrid data were evaluated to analyze the effect of gender on person re-identification. The smartphone data include a male to female ratio of 37:23. To analyze the effect of gender, the models were trained and tested for each gender separately. For the step data, the classification accuracies of female and male subjects were 86.75% and 85.71%, respectively. The classification accuracies for stride data were slightly better than those of the step data. The classification accuracies were 87.25% for male group and 90.12% for female group. The results are shown in Table 10 and Figure 16.

#### 4.3.3. Applying Gender Restrictions on Wearable Data

The female to male ratio in wearable data is 14:12. Table 11 shows the classification accuracies of male and female with reference to step and stride data. The observed accuracies remained above 99% on step data while 100% on stride data. For visual difference in age-groups, the bar graphs are shown in Figure 16.

## 5. Discussion

**Figure 17:**
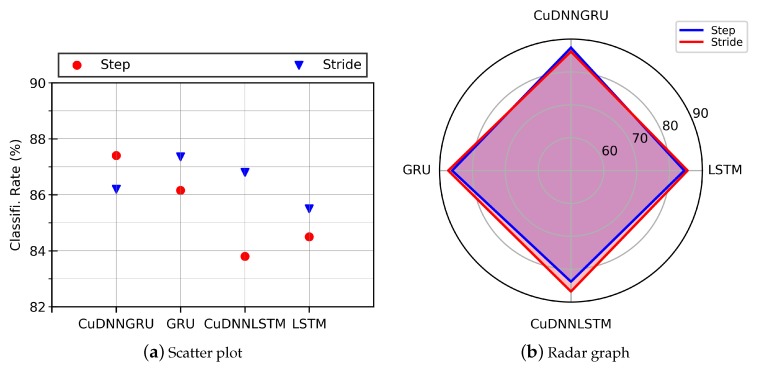
(**a**) Scatter plot; and (**b**) radar graph show the performance of models trained and tested on our gait data. The results show higher accuracies for the stride dataset (200 timesteps) than the step dataset (100 timesteps).

**Figure 18:**
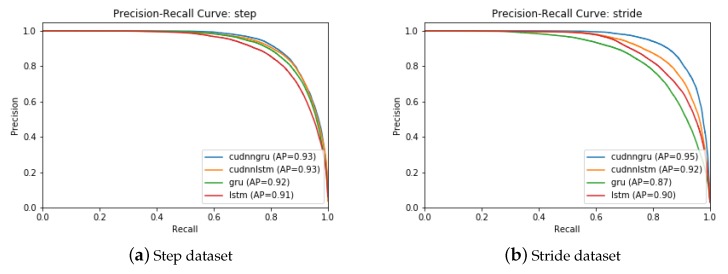
Precision–recall graphs computed by four different deep models (CuDNNGRU, GRU, CuDNNLSTM, and LSTM) from: (**a**) step data; and (**b**) stride data. CuDNNGRU outperformed the rest of the models in all cases.

The objective of the work was to recognize and identify a person using the inertial gait data of normal human walk collected by means of body mounted sensors. For this purpose, the inertial gait data collected in one of our previous work [8] were used.The data were obtained using 6D accelerometer and gyroscope embedded on a smartphone and wearable device at a sampling rate of 75 Hz. The sensors were mounted on the body of the subjects at chest position. The dataset is composed of data from 86 individuals and the data were recorded under varying conditions such as varying surface types and their frictions, subjects trying to fake their gait, etc. By exploiting the fact that the stepping frequency of normal human walk remains within 1–2 Hz, we segmented the low-level input signals into 100 Hz to extract steps and into 200 Hz to extract strides. A stride is characterized as consecutive heel strikes of the same foot, whereas a step is described as the heel strike of one foot followed by that of the other foot (alternation of heel strikes). Both datasets, i.e., step data and stride data, were used to train four deep learning models: *GRU*, *CuDNNGRU*, *LSTM*, and *CuDNNLSTM*. In general, CudNNGRU outperformed the rest of the deep learning models by re-identifying persons with higher accuracies, as shown in Figure 17. The precision–recall graphs computed from the different deep learning models using step and stride data are shown in Figure 18, where it is observable that CuDNNGRU outperformed the rest of the models. The results were computed on hybrid data (where data collected with wearable’s IMU and smartphone’s IMU were mixed), smartphone’s IMU data, and wearable’s IMU data. The results show that above 86% of the subjects were accurately re-identified in Rank-1 and above 95% subjects were accurately re-identified in Rank-5 for all three datasets and for both models trained with the step data or the stride data, respectively. The trend remained the same when age and gender restrictions were applied to the aforementioned datasets. In terms of comparison of the classification accuracies achieved with the model trained over the step data and the model trained over the stride data, the latter model outperformed the former in most cases.

**Figure 19:**
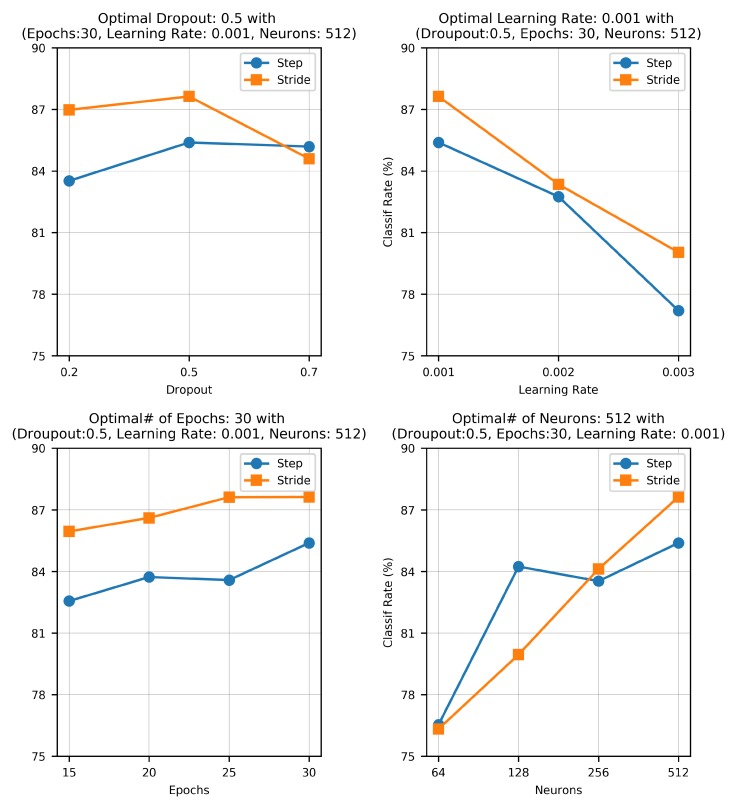
The effect of different hyperparameters including size of the network (specified by the number of neurons), drop-out rate, learning rate, and number of epochs are shown here. The optimal network parameters were 512 neurons trained for 30 epochs with a learning rate of 0.001 and a dropout value of 0.5.

**Figure 20:**
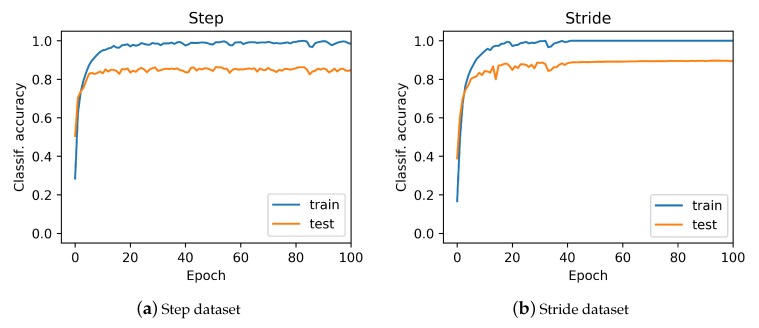
The relationship between the network recognition effect and its training times from: (**a**) step data; and (**b**) stride data. The model was trained for 100 epochs.

To optimize the model performance, we tuned and tested the network under different hyperparameter settings. In particular, we varied four basic hyperparameters: the size of the network (specified by the number of neurons), the drop-out rate, the learning rate, and the number of epochs. The results of this ablation study are shown in Figure 19. Here, we see that the model’s performance increased (i.e, testing accuracy improved) by increasing the size of the network and the number of training epochs. By further increasing the number of neurons and additional layers, we observed a relative dip in model accuracy. Such a degraded performance was probably due to the well-known vanishing gradient problem. In addition, by increasing the size of the network, the model started to overfit to the training data owing to the growth in network parameters. The optimal network parameters obtained after number of trials are 512 number of neurons trained for 30 epochs with a learning rate of 0.001 and a dropout value of 0.5. The relationship between the network recognition effect and its training times is shown in Figure 20. The model was trained for 100 epochs.

Moreover, we also compared the results with other deep neural network architectures such as convolutional neural network (CNN). We used the same parameters settings but the obtained testing accuracy was found to be lower as compared to the sequential CuDNNGRU architecture. These comparison accuracies of both step and stride using hybrid data are reported in Table 12. Furthermore, one could also train conventional machine learning classifiers such as support vector machines instead of deep neural network architectures but that requires the tedious and challenging task of feature engineering, which often lacks the generalization capabilities. Owing to these well-known drawbacks, we opted to bypass such hand-crafted feature extraction with the automatic feature learning (i.e., representational learning) ability of deep recurrent neural network-based architectures and therefore selected them for training/learning in this work.

The proposed system gives us several interesting insights. It has the potential to physically track subjects, i.e., if the system has already seen the gait patterns of a particular subject, then it can easily re-identify the subject based on the learned gait signatures. Furthermore, the results on sensor modalities show that, once the gait signatures of a subject are exposed and learned by the system, the system can reliably track the subject despite a change in the body mounted sensor, e.g., replacing a wearable IMU with a smartphone. Re-identification and tracking of a subject by the proposed system also raises serious privacy concerns as the system has the ability to track subjects solely based on the inertial signatures of their gait.

## 6. Conclusions and Future Work

The proposed work is independent of the challenges that occur in vision-based techniques, i.e., occlusion, illumination, view point variation, camera calibration, etc. Human gait is believed to be unique [6,53], therefore the walking signatures of human gait can be used for human identification. The proposed approach learns and exploits this uniqueness in human gait signatures, which works regardless of the device a subject is carrying provided that it has on-board inertial sensors. We extensively tested the uniqueness in human gait signatures under different experimental setups using the data collected with smartphones and wearables (see Section 4.1). The results show that persons can be re-identified with high accuracy and confidence based on the analysis of their gait. In addition to that, the higher classification accuracies of the proposed architecture shows that human gait is a suitable candidate for identification. In the future, the model can be extended for solving open-world re-identification in real time, where the system will identity the already existing subjects as well as new subjects, who are new in the open loop. Person identification, tracking, and surveillance at public places by utilizing inertial sensors of smartphones and wearables are the possible future application areas. We can also build a multi-modality system by fusing inertial sensors with vision sensor.

The results obtained with the current experimental set up are only for the sake of proof of concept. There exist several challenging open areas which need to be addressed to develop an adoptable solution. A key limitation of the proposed work is statistically unbalanced population where there are variations in male to female ratio, age groups, ground surfaces, etc. In the future, we will extend the datasets to minimize the aforementioned variations in the population and will test the proposed approach with a larger database. Another important limitation of the work is unbalanced data as only approximately 33% of the data were collected with the wearable sensor. Collection of more data with the wearable sensors is another direction of the future work. The sensor placement, which was firmly fixed on the chest of the subjects, is another notable restriction in the proposed work. A practical direction, which is still an open challenge, would be collecting data with a wearable sensor regardless of its orientation and its use for person re-identification.

## Figures and Tables

**Table 1:** Characteristics of the population. The data were collected by Riaz et al. [8] under three different setups: Setups A and B using smartphone’s on-board IMU (MPU-6500) and Setup C using the APMD Opal IMU.

	All Setups	Setup A	Setup B	Setup C
**Participants**	86	40	20	26
**Male:Female**	49:37	26:14	11:9	12:14
**Age (years, μ±σ)**	33.8 ± 14.8	25.2 ± 5.9	32.6 ± 13.7	48.1 ± 12.7
**Height (cm, μ±σ)**	172.1 ± 8.9	171.6 ± 8.4	169.8 ± 7.9	174 ± 10.2

**Table 2:** The ranking results along with mean average precision computed with the steps and the strides hybrid data are shown. For both steps and strides, above 86% of the subjects were correctly re-identified in Rank-1, whereas above 97.50% of the subjects were correctly re-identified in Rank-5.

	Rank-1 (%)	Rank-5 (%)	Rank-10 (%)	Rank-20 (%)	mAP (%)
**Step**	86.23	98.36	99.6	99.91	90.33
**Stride**	87.15	97.89	99.16	99.70	91.77

**Table 3:** The ranking results and mean average precision computed with the steps and the stride data collected using the smartphone’s IMU are shown. For both steps and strides, above 86% of the subjects were correctly re-identified in Rank-1, whereas above 98% of the subjects were correctly re-identified in Rank-5.

	Rank-1 (%)	Rank-5 (%)	Rank-10 (%)	Rank-20 (%)	mAP (%)
**Step**	86.18	98.13	99.05	99.93	91.36
**Stride**	86.95	98.09	99.02	99.07	91.09

**Table 4:** The ranking results along with mean average precision computed from the steps and the stride data collected using wearable IMU are shown here. For both steps and strides, above 98% of the subjects were correctly re-identified in Rank-1, whereas above 99% of the subjects were correctly re-identified in Rank-5.

	Rank-1 (%)	Rank-5 (%)	Rank-10 (%)	Rank-20 (%)	mAP (%)
**Step**	98.06	99.96	99.99	100.00	99.26
**Stride**	98.73	99.98	100.00	100.00	99.34

**Table 5:** The subjects participated in the data collection sessions are placed in six age groups. The number of subjects in each age group under different datasets, i.e., hybrid, smartphone, and wearable IMU, are shown.

		No. of Subjects		
	Age Groups (y)	Hybrid	Smartphone	Wearable IMU
G${}_{1}$	10–19	3	3	0
G${}_{2}$	20–29	48	47	1
G${}_{3}$	30–39	9	1	8
G${}_{4}$	40–49	10	4	6
G${}_{5}$	50–59	9	4	5
G${}_{6}$	60–69	7	1	6

**Table 6:** Results of test accuracies computed with the models trained on steps and strides hybrid data when applying age restriction are shown here. The numbers in parentheses indicate the number of subjects in the respective group.

	Test Accuracies (%)					
	G${}_{1}$ (3)	G${}_{2}$ (48)	G${}_{3}$ (9)	G${}_{4}$ (10)	G${}_{5}$ (9)	G${}_{6}$ (7)
**Step**	93.03	83.58	90.53	89.13	97.23	97.59
**Stride**	98.57	85.55	94.31	98.07	97.36	100.00

**Table 7:** Results of test accuracies computed with the models trained on steps and strides smartphone’s IMU data when applying age restriction are shown here. The numbers in parentheses indicate the number of subjects in the respective group.

	Test Accuracies (%)					
	G${}_{1}$ (3)	G${}_{2}$ (47)	G${}_{3}$ (1)	G${}_{4}$ (4)	G${}_{5}$ (4)	G${}_{6}$ (1)
**Step**	90.74	84.17	98.55	93.53	94.82	96.43
**Stride**	98.59	85.86	96.43	95.83	100.00	100.00

**Table 8:** Results of test accuracies computed with the models trained on step and stride wearable IMU data when applying age restriction are shown here. The numbers in parentheses indicate the number of subjects in the respective group.

	Test Accuracies (%)				
	G${}_{1}$ (1)	G${}_{2}$ (8)	G${}_{3}$ (6)	G${}_{4}$ (5)	G${}_{5}$ (6)
**Step**	98.97	100.00	93.53	98.55	100.00
**Stride**	100.00	100.00	95.83	100.00	100.00

**Table 9:** This table shows that, using step and stride data, the accuracy of female subjects was about 1% greater than male subjects.

	Test Accuracies (%)	
	Male (49)	Female (37)
**Step**	85.67	86.50
**Stride**	88.34	89.12

**Table 10:** This table shows the accuracies of *37* male and *23* female subjects.

Test Accuracies (%)		
	Male (37)	Female (23)
**Step**	85.71	86.75
**Stride**	87.25	90.12

**Table 11:** This table shows the re-identification accuracies of *12* male and *14* female subjects. The accuracy was above 99% on step data while 100% on stride data.

Test Accuracies (%)		
	Male (12)	Female (14)
**Step**	99.32	99.36
**Stride**	100.00	100.00

**Table 12:** Results comparison of CuDNNGRU with Convolutional Neural Network (CNN) using same parameters.

Accuracy	CuDNNGRU	CNN
Step	87.15%	69.05%
Stride	86.23%	76.04%
